# Obesity treatment effect in Danish children and adolescents carrying *Melanocortin-4 Receptor* mutations

**DOI:** 10.1038/s41366-020-00673-6

**Published:** 2020-09-13

**Authors:** Cæcilie Trier, Mette Hollensted, Theresia M. Schnurr, Morten Asp Vonsild Lund, Tenna Ruest Haarmark Nielsen, Gao Rui, Ehm Astrid Andersson, Mathilde Svendstrup, Dorthe Sadowa Bille, Anette P. Gjesing, Cilius Esmann Fonvig, Christine Frithioff-Bøjsøe, Marie Balslev-Harder, Shi Quan, Michael Gamborg, Oluf Pedersen, Lars Ängquist, Jens-Christian Holm, Torben Hansen

**Affiliations:** 1 Novo Nordisk Foundation Center for Basic Metabolic Research, Faculty of Health and Medical Sciences, University of Copenhagen, Copenhagen, Denmark; 2 The Children’s Obesity Clinic, Department of Pediatrics, Copenhagen University Hospital Holbæk, Holbæk, Denmark; 3 Department of Pediatrics, Copenhagen University Hospital Rigshospitalet, Copenhagen, Denmark; 4 Danish Diabetes Academy, Odense, Denmark; 5 Department of Biomedical Sciences, Faculty of Health and Medical Sciences, University of Copenhagen, Copenhagen, Denmark; 6 Geneplus, Bejing Institute, Bejing, China; 7 Department of Pediatrics, Kolding Hospital a part of Lillebælt Hospital, Kolding, Denmark; 8 BGI-Shenzhen, Shenzhen, China; 9 Ferring Pharmaceuticals, Copenhagen, Denmark; 10 The Faculty of Health and Medical Sciences, University of Copenhagen, Copenhagen, Denmark

**Keywords:** Epidemiology, Genetics

## Abstract

**Objectives:**

To determine the prevalence of *Melanocortin-4 Receptor* (*MC4R*) mutations in a cohort of children and adolescents with overweight or obesity and to determine whether treatment responses differed between carriers and noncarriers.

**Methods:**

Using target region capture sequencing, an *MC4R* mutation screen was performed in 1261 Danish children and adolescents enrolled at a tertiary multidisciplinary childhood obesity treatment center. Measurements of anthropometrics, blood pressure, fasting blood biochemistry including lipid and hormone levels, and dual-energy X-ray absorptiometry were performed at baseline and throughout treatment.

**Results:**

Of 1209 children and adolescents that met all criteria to be included in the described analyses, 30 (2.5%) carried damaging or unresolved *MC4R* mutations. At baseline, mutation carriers exhibited higher concentrations of plasma thyroid-stimulating hormone (*p* = 0.003), and lower concentrations of plasma thyroxine (*p* = 0.010) compared to noncarriers. After a median of 1 year of treatment (range 0.5–4.0 years), body mass index (BMI) standard deviation score (SDS) was reduced in noncarriers but not in carriers, and this difference in treatment response was statistically significant (*p* = 0.005). Furthermore, HDL cholesterol was reduced in carriers, a response significantly different from that of noncarriers (*p* = 0.017).

**Conclusion:**

Among Danish children and adolescents with overweight or obesity entering a tertiary lifestyle intervention, 2.5% carried damaging or unresolved *MC4R* mutations. In contrast to noncarriers, carriers of damaging or unresolved *MC4R* mutations failed to reduce their BMI SDS during obesity treatment, indicating a need for personalized treatment based on the *MC4R* genotype.

## Introduction

The *Melanocortin-4 Receptor* (*MC4R*) is expressed throughout the brain, especially in the hypothalamus, and the encoded protein regulates appetite and energy expenditure [1]. The number of identified *MC4R* mutations exceeds 369 [2, 3], and *MC4R* deleterious mutations are responsible for an autosomal co-dominant form of monogenic obesity [4]. While the prevalence of carriers of *MC4R* mutations varies with ethnicity [5–7], up to 2% carry damaging mutations in *MC4R* among samples of non-consanguineous individuals with obesity of European descent, making MC4R deficiency the most common form of monogenic obesity [4]. Based on studies including children with damaging *MC4R* mutations, specific characteristics have been associated with MC4R deficiency, namely increased fat and lean mass, increased linear growth, increased bone mineral density, hyperphagia, and hyperinsulinemia [8, 9]. In addition, individuals deficient of MC4R appear to have lower blood pressure, lower heart rate, and a lower prevalence of hypertension compared to noncarriers [10]. However, the above phenotype characteristics of carriers of damaging *MC4R* mutations remain controversial as other studies have failed to replicate them [11–15].

Few studies investigated the possible differences in response to obesity treatment between *MC4R* mutation carriers and noncarriers in children. Generally, the mutations do not seem to influence the effect of lifestyle interventions [16–18], though carriers of an *MC4R* mutation may have difficulties maintaining their weight loss [16]. These intervention studies have been limited by small study samples and short duration. It therefore remains unclear whether carriers of *MC4R* mutations respond differently to a structured longitudinal lifestyle intervention targeting obesity when compared to noncarriers.

The Children’s Obesity Clinic (TCOC) is the largest clinic in Denmark offering validated childhood obesity treatment, and the clinic has shown solid treatment results [19]. Treatment results in patients carrying *MC4R* mutations have not yet been investigated. Thus, the aim of the present study was to: (1) determine the prevalence of carriers of damaging or unresolved *MC4R* mutations in a cohort of children and adolescents with overweight or obesity participating in a structured longitudinal lifestyle intervention at TCOC, (2) describe the clinical characteristics of carriers with obesity and compare them to noncarriers, and (3) assess differences in weight loss intervention response between carriers and noncarriers by analyzing changes in anthropometrics, body composition, and metabolic traits.

## Methods and materials

### Study population

We recruited 1261 Danish children and adolescents aged 2–19 years through TCOC (Department of Pediatrics, Copenhagen University Hospital Holbæk, Denmark). Patients were included into TCOC Data- and Biobank between January 2009 and June 2014 and followed until December 2016. Exclusion criteria were a body mass index (BMI) below the ~90th percentile according to age and sex (BMI *z*-score < 1.28) in a Danish reference [20] (*n* = 5), missing baseline date (*n* = 1), baseline age below 2 years (*n* = 1), or above 19 years (*n* = 16). Siblings to previously included patients were also excluded for the present study (*n* = 29). *MC4R* mutations were screened among all patients.

The current study was performed in accordance with the Helsinki Declaration, and informed written consent was obtained from parents/guardians or from participants aged 18 years and older. The study was approved by the Danish Data Protection Agency, the Ethics Committee of the Region Zealand in Denmark (ID-no. SJ-104) and was registered at ClinicalTrials.gov (ID-no. NCT00928473).

### Clinical examination

Anthropometric data, blood samples, and dual-energy X-ray absorptiometry (DXA) scans were obtained at the primary visit (baseline). During treatment, anthropometric measurements were ideally performed at each visit, while blood samples and DXA were ideally obtained annually.

Weight was measured to the nearest 0.1 kg, and height was measured to the nearest 1 mm [19]. BMI standard deviation score (SDS) and height-for-age SDS were calculated using Danish references [20].

Blood pressure was measured on the upper right arm after 5 min of rest [19]. Systolic blood pressure (SBP) and diastolic blood pressure (DBP) SDS were calculated according to guidelines from the European Society of Hypertension [21], and compared to the distribution in an American reference [21, 22]. These values were transformed into SDSs (*z-*scores).

Fasting venous blood samples included hemoglobin A1c (HbA1c), plasma concentrations of glucose, thyroid-stimulating hormone (TSH), triiodothyronine (T3), thyroxine (T4), total cholesterol, low-density lipoprotein (LDL) cholesterol, high-density lipoprotein (HDL) cholesterol, and triglycerides, as well as serum concentrations of insulin.

DXA measurements of total body mass, total body fat mass, and bone mineral density (BMD) were performed with a GE Lunar Prodigy (DF+10031, GE Healthcare, Little Chalfont, United Kingdom) until 14 October 2009 and thereafter on a GE Lunar iDXA (ME+200179, GE Healthcare, Little Chalfont, United Kingdom). Body fat mass index (BFMI) and fat-free mass index (FFMI) were calculated as fat mass or fat-free mass in kilograms divided by height in meters squared [23].

Trained pediatricians assessed pubertal development stages according to the methods of Tanner [24, 25]. For *n* = 278 out of *n* = 1209 participants a pubertal measure was unavailable (23.0%), and for them, we imputed stages sex-specifically by ordered logistic regression—based on the initial five stages—adjusted for age, height, height-squared and HDL cholesterol (the latter was initially imputed for a few observations based on linear regression adjusted for age, sex, BMI, and height).

At baseline, when the interval between the date of the clinical examination and the date of the blood sample or DXA scan exceeded 60 days, measurements for the respective participant were not included in the analyses. Furthermore, outliers (individual minimum/maximum values located far from the next value; checked iteratively) were excluded prior to analyses (Tables 1 and 2).

**Table 1:** Baseline characteristics of Danish children and adolescents with and without damaging *MC4R* mutations enrolled into obesity treatment.

	Children with damaging or unresolved *MC4R* mutations (group A)		Children with benign or no *MC4R* mutations (group B)		*p* for difference between groups A and B^a^	
	*n*	Mean (SD) or %	*n*	Mean (SD) or %		Adjusted for BMI
Sex, % boys	30	46.7	1179	44.3	0.78	0.92
Age, years	30	10.28 (3.68)	1179	11.62 (3.08)	**0.019**	**0.022**
BMI SDS	30	3.08 (0.71)	1179	2.98 (0.69)	0.80	–
Height-for-age SDS	30	1.18 (1.10)	1179	0.92 (0.98)	0.41	0.43
FFMI, kg/m^2^ (log)	21	2.70 (0.16)	865	2.72 (0.14)	0.25	0.16
BFMI, kg/m^2^ (log)	21	2.32 (0.26)	865	2.41 (0.28)	0.80	0.46
BMD, g/cm^2^ (log)	21	−0.065 (0.15)	865	−0.023 (0.15)	0.58	0.59
SBP SDS	28	0.73 (0.98)	1091	0.70 (1.00)	0.94	0.97
DBP SDS	28	−0.027 (0.97)	1091	0.21 (0.78)	0.063	0.057
Glucose, mmol/l	21	5.29 (0.40)	991	5.19 (0.57)	0.24	0.24
HbA1c, mmol/mol	23	35.04 (4.62)	1032	34.80 (3.62)	0.70	0.71
Insulin, pmol/l (log)	23	4.67 (0.47)	1041	4.76 (0.63)	0.97	0.97
TSH, mU/l (log)	23	1.30 (0.72)	1039	0.98 (0.50)	**0.004**	**0.004**
T3, nmol/l	23	2.36 (0.41)	1035	2.41 (0.39)	0.44	0.44
T4, pmol/l^b^	23	14.55 (2.54)	1036	15.29 (2.05)	**0.023**	**0.022**
Total cholesterol, mmol/l^b^	23	4.49 (0.78)	1028	4.22 (0.77)	0.13	0.13
HDL cholesterol, mmol/l	23	1.24 (0.25)	1029	1.22 (0.29)	0.98	0.99
LDL cholesterol, mmol/l^b^	23	2.70 (0.81)	1026	2.51 (0.67)	0.22	0.22
Triglycerides, mmol/l (log)	23	0.074 (0.46)	1028	−0.037 (0.51)	0.18	0.18

We present mean and standard deviation for each of the two groups (groups A and B), while presenting *p* values (without and with adjustment for BMI) for the mean difference between groups A and B at baseline. *p* values in bold indicate *p* < 0.05.

*MC4R* Melanocortin-4 Receptor, *BMI* body mass index, *SDS* standard deviation score, *FFMI* fat-free mass index, *BFMI* body fat mass index, *BMD* bone mineral density, *SBP* systolic blood pressure, *DBP* diastolic blood pressure, *HbA1c* hemoglobin A1c, *TSH* thyroid-stimulating hormone, *T3* triiodothyronine, *T4* thyroxine, *HDL* high-density lipoprotein, *LDL* low-density lipoprotein.

^a^Data were analyzed with a multiple regression analysis, except sex difference by logistic regression (adjusted for age). Analyses were adjusted as appropriate for age, sex, pubertal development stage, and type of DXA scanner (age only adjusted for sex). We also present all significance results from analyses with additional adjustment for BMI.

^b^Outliers excluded prior to analyses (*n* = 1 in group B for T4, total cholesterol, and LDL cholesterol).

**Table 2:** Changes in traits after lifestyle intervention in Danish children and adolescents with and without damaging *MC4R* mutations.

	Children with damaging or unresolved *MC4R* mutations (group A)		Children with benign or no *MC4R* mutations (group B)		*p* for difference of mean delta change between groups A and B^a^	
	*n*	Mean delta change (SD)	*n*	Mean delta change (SD)		Adjusted for BMI change
BMI SDS	24	−0.033 (0.45)	982	−0.27 (0.43)	**0.005**	–
Height-for-age SDS	24	−0.097 (0.21)	982	−0.025 (0.29)	0.38	0.44
FFMI, kg/m^2^ (log)	13	0.081 (0.067)	555	0.049 (0.063)	0.15	0.41
BFMI, kg/m^2^ (log)	13	0.11 (0.23)	555	−0.041 (0.21)	0.13	0.73
BMD, g/cm^2^ (log)	13	0.035 (0.046)	555	0.041 (0.059)	0.090	0.057
SBP SDS	19	−0.44 (0.94)	792	−0.16 (1.06)	0.19	0.080
DBP SDS	19	0.17 (0.87)	792	−0.20 (0.84)	0.39	0.72
Glucose, mmol/l	8	−0.1 (0.53)	549	0.041 (0.66)	0.82	0.84
HbA1c, mmol/mol^b^	11	−0.18 (2.18)	581	−0.29 (2.30)	1.00	0.87
Insulin, pmol/l (log)	12	0.11 (0.65)	569	−0.22 (0.67)	0.18	0.50
TSH, mU/l^b^ (log)	11	−0.21 (0.52)	589	−0.10 (0.42)	0.72	0.79
T3, nmol/l	10	0.005 (0.27)	461	−0.041 (0.36)	0.79	0.55
T4, pmol/l^b^	11	−0.045 (2.90)	585	−0.84 (2.29)	0.69	0.56
Total cholesterol, mmol/l^b^	11	−0.21 (0.76)	577	−0.066 (0.60)	0.72	0.55
HDL cholesterol, mmol/l	11	−0.11 (0.19)	579	0.047 (0.22)	**0.017**	**0.050**
LDL cholesterol, mmol/l^b^	11	−0.18 (0.71)	576	−0.11 (0.53)	0.97	0.77
Triglycerides, mmol/l^b^ (log)	11	0.13 (0.77)	576	−0.0074 (0.46)	0.24	0.44

When analyzing the lifestyle intervention response outcomes, mean and standard deviation with respect to change from baseline (delta change) were calculated for each group. We then assessed the difference between the mean values between groups A and B (hence comparing differences-in-differences). *p* values in bold indicate *p* < 0.05.

*MC4R* Melanocortin-4 Receptor, *CI* confidence interval, *BMI* body mass index, *SDS* standard deviation score, *SBP* systolic blood pressure, *DBP* diastolic blood pressure, *FFMI* fat-free mass index, *BFMI* body fat mass index, *TSH* thyroid-stimulating hormone, *HDL* high-density lipoprotein, *LDL* low-density lipoprotein.

^a^Data were analyzed with a multiple regression analysis adjusted for age, sex, pubertal status, baseline value of the respective trait, follow-up time, and type of DXA scanner (where appropriate). We also present all significance results from analyses with additional adjustment for BMI change.

^b^Outliers excluded prior to analyses (*n* = 1 in group B for T4, total cholesterol, LDL cholesterol, and triglycerides; *n* = 2 in group B for HbA1c and TSH).

### *MC4R* mutation screen

All participants were screened for *MC4R* mutations using a target region capture sequencing platform [26]. In brief, genomic DNA extracted from peripheral blood lymphocytes was fragmented and purified, and A-tailing was added after blunt end repair. Fragments were ligated and amplified through a polymerase chain reaction (PCR) using paired-end primers containing index tags. Purified PCR products were then hybridized to a GenCap™ probe and washed. Subsequently, captured DNA libraries were amplified through PCR and sequenced using the Illumina HiSeq2000 Analyzers, providing a ≥150-fold average depth for >95% of samples. Reads were aligned to the GRCh37/hg19 human reference genome (UCSC Genome Browser) using the Burrows-Wheeler Aligner (BWA, v.0.6.2). All variants were called by HaplotypeCaller from GATK (https://www.broadinstitute.org/gatk/) using de novo assembly of haplotypes in the target region, and filtering of variants was performed using the GATK tool Variant Filtration.

Potential mutations were then selected if: (1) their allele frequency was below 0.01% in publicly available variant databases (Exome Variant Server: http://evs.gs.washington.edu/EVS/; dbSNP: http://www.ncbi.nlm.nih.gov/SNP/; 1000G: http://browser.1000genomes.org/index.html; and ExAC Browser: http://exac.broadinstitute.org/), and (2) the variation resulted in: loss or alteration of start or stop codons, alterations in the open reading frame, alterations in or near splice sites, missing transcripts, or other changes in the protein codon. Except for *MC4R*, no other genes were analyzed.

### Classification of mutations

The potential phenotypic effect of identified variants was assessed using previously published in vitro functional studies. Based on these, variants were classified as either “damaging,” “unresolved,” or “benign” clinical significance. A variant was considered damaging if it had repeatedly been found to significantly impair *MC4R* expression or normal MC4R function and activity in vitro. We defined two groups of children, namely children with damaging or unresolved *MC4R* mutations (hereafter termed group A) and children with benign or no *MC4R* mutations (hereafter termed group B) (Table 2). While our primary analysis is based on the comparison between these two groups (Tables 1 and 2), we performed additional sensitivity analysis excluding children with benign *MC4R* mutations from group B.

### Intervention

TCOC encompasses a full multidisciplinary best-practice tertiary team including pediatricians, nurses, dietitians, psychologists, research technicians, social workers, and secretaries. At baseline, the child/adolescent and family receive a treatment plan comprising 10–25 advices, including attention to disturbed eating behaviors, physical activity and inactivity, sources and amounts of nutrition, psychosocial functioning, sleep patterns etc. At each visit, the plan is modified according to the needs of, and in collaboration with, the individual child or adolescent and his/her family. As treatment progresses the frequency and type of visits are equally adjusted to best support individualized treatment. The treatment protocol has previously been extensively described [19]. TCOC has reported reductions in BMI SDS in 75% of patients after 1 year of treatment [27]. On average, 5.4 h were invested in each patient per year with consultations every 6–8 weeks [19].

We selected the available follow-up observation, if any, for the individual patient that was closest to 1 year from the baseline observation; only follow-up times within the range from 6 months to 4 years were accepted. This was done separately for anthropometric variables, blood pressure variables, blood-sample variables, and DXA variables. In addition, we required an available BMI measure within 60 days from the follow-up date, with the exception that—if no BMI observation was found within these 60 days—DXA variables were allowed to use measured BMI from the corresponding DXA visit.

### Statistical analysis

After excluding 52 children and adolescents as listed above, we included the remaining 1209 children and adolescents with eligible baseline data in our analysis. We performed all statistical analyses with and without inclusion of individuals with Down’s syndrome, severe mental retardation, genetic disorders affecting growth, chromosome abnormalities, diabetes mellitus type I, as well as children receiving regular treatment with orally or intravenously administered steroid hormones (*n* = 27 children, including two carriers of damaging *MC4R* mutations; total sample size *n* = 1182). Since inclusion of these individuals virtually did not influence the results, only the results obtained in the full data set are provided. Prior to statistical analyses, non‐normally distributed traits at baseline (BFMI, FFMI, BMD, plasma concentrations of TSH and triglycerides, and serum concentrations of insulin) were logarithmically transformed to comply with the assumption of normal distribution.

At baseline, differences in age and sex were assessed by linear regression (adjusted for sex) and logistic regression (adjusted for age), respectively. Traits previously associated with MC4R deficiency, other quantitative metabolic traits, and anthropometric measures were tested for differences between carriers of *MC4R* mutations and noncarriers using multiple regression analysis. Analyses were adjusted for age, sex, pubertal stage (three levels: prepubertal (Tanner I), peripubertal (Tanner II–IV), postpubertal (Tanner V)), and type of DXA scanner (where appropriate). We also performed analysis with additional adjustment for BMI.

When analyzing the lifestyle intervention response outcomes, mean and standard deviation with respect to change from baseline were calculated for each group and the differences between the mean values between carriers of *MC4R* mutations and noncarriers were assessed (hence comparing differences-in-differences). These analyses were adjusted for baseline age, sex, pubertal status, baseline value of the respective trait, follow-up time, and type of DXA scanner (where appropriate). We also performed analyses with additional adjustment for BMI change. While we report the results for the overall changes during the follow-up period, we also calculated and used the changes scaled to 1-year changes and observed that results did not differ substantially (data not shown).

All statistical analyses were performed using Stata version 15.1 (StataCorp LLC, College Station, TX; www.stata.com).

To acknowledge the multiple tests being performed, and to add another layer of interpretation, we also derived multiple testing-corrected *p* values. When calculating the *p* value correction factor, we considered that two independent questions were asked. Namely, baseline phenotypic differences between carriers and noncarriers, as well as the difference of response to the lifestyle intervention between carriers and noncarriers. Hence, we applied a separate Bonferroni correction for both questions (based on the correction factor *n* = 17, interpreting baseline age and sex as background variables, see Tables 1 and 2).

## Results

### Identification of damaging *MC4R* mutations

Among the 1209 children, targeted sequencing of *MC4R* enabled the identification of 13 non-silent variants among 101 carriers. Two of these variants, the nonsense mutation Y35X and the nucleotide substitution 110A>T, constitute a haplotype and were found in the same 12 individuals. All variants were heterozygous and have previously been described (Table 3).

**Table 3:** Identified *MC4R* variants among 1261 Danish children and adolescents with overweight or obesity.

Type of mutation	AA change	Nucleotide change	SNP ID	Carriers (*n*)	In vitro functional analyses	
					Functional effect	References
Classified as “damaging” (total number of carriers with damaging mutation: *n* = 25)						
Nonsense	p.Y35X and p.D37V	c.105C>A ac.110A>T	rs13447324 rs13447325	12^a^	Reduced ligand-induced cAMP signaling, reduced cell-surface expression	[28, 29, 32, 35]
Missense	p.R165Q	c.494G>A	NA	8	Reduced ligand-induced cAMP and ERK1/2 signaling	[8, 29, 30, 32, 33, 35, 55]
					Loss of function	[34]
Missense	p.G181D	c.542G>A	rs13447333	1	Loss of function, reduced cell-surface expression	[14, 28, 31, 35]
Missense	p.A219V	c.656C>T	rs121913567	4	Reduced ligand-induced cAMP signaling, reduced basal pERK1/2 levels	[31, 55–57]
Classified as “unresolved” (total number of carriers with unresolved mutation: *n* = 6)						
Missense	p.T112M	c.335C>T	rs13447329	2	Cell-surface expression, ligand binding, and cAMP accumulation as WT	[14, 28, 37–39, 41]
					Reduced cell-surface expression, impaired ligand-stimulated ERK1/2 activation	[34, 55]
					Reduced potency for some endogenous ligands	[29]
Missense	p.I170V	c.508A>G	rs121913560	3^a^	Ligand-induced cAMP and ERK1/2 signaling as WT	[29, 42, 55, 58]
					Reduced ligand-induced cAMP signaling, reduced cell-surface expression	[13, 34, 40, 58]
Missense	p.V253I	c.757G>A	rs187152753	1	As WT	[29, 34, 40]
					Reduced ligand-induced cAMP signaling	[33]
Classified as “benign” (total number of carriers with benign mutation: *n* = 71)						
Missense	p.S30F	c.89C>T	rs13447323	1	As WT, agonist-stimulated ERK1/2 activation	[4, 28, 30, 38, 40, 43, 55]
Missense	p.M200V	c.598A>G	NA	2	Cell-surface expression, ligand-induced cAMP and ERK1/2 signaling as WT	[14, 36, 37, 55]
Missense	p.P275S	c.823C>T	rs201813179	1	As WT	[32]
Missense	p.V103I	c.307G>A	rs2229616	38	As WT	[13, 14, 28, 32, 39]
					Gain of function	[29]
Missense	p.I251L	c.751A>C	rs52820871	29	As WT	[13, 14, 28, 32]
					Gain of function	[29]

Identified *MC4R* variants, their overall classification in this study and their corresponding change in amino acid composition and nucleotides. For each identified variant, SNP ID and number of carriers are listed. Functional effects, as examined by in vitro analyses, are summarized and cited with references; based on this, the overall classification of each variant is provided.

*AA* amino acid, *ERK1/2* extracellular signal-regulated kinases 1 and 2, *SNP* single nucleotide polymorphism.

^a^One child carried both the p.Y35X and p.D37V/ac.110A>T haplotype and the p.I170V variant.

The functionality of all identified variants was assessed based on previous in vitro functional studies, and an overview is provided in Table 3. Of the identified *MC4R* mutations, four (Y35X/110A>T, R165Q, G181D, and A219V) were classified as damaging [8, 28–35]. Four variants (M200V, V103I, I251L, and P275S, S30F), previously found not to reduce MC4R activity in vitro [13, 28, 29, 32, 36, 37], were classified as benign. Previous in vitro functional studies on three variants (T112M, I170V, and V253I) have shown inconsistent results with a few studies reporting damaging effects and others reporting no reduction in MC4R activity [13, 28, 29, 33, 34, 37–43]. These variants were classified as of unresolved clinical significance. Based on this classification, we identified 30 children with damaging or unresolved *MC4R* mutations (prevalence of damaging or unresolved *MC4R* mutations: 30/1209 = 2.5%, group A). One child carried both the damaging haplotype Y35X/110A>T and the I170V variant classified as unresolved. Overall, 71 children were carriers of mutations, which were of benign clinical significance (Table 3) and were included in the group of patients without *MC4R* mutations, comprising 1179 individuals (group B).

### Effects of damaging or unresolved *MC4R* mutations at baseline

Clinical and biochemical baseline characteristics for the 30 children with damaging or unresolved *MC4R* mutations (group A) and the 1179 patients with benign or without *MC4R* mutations (group B) are provided in Table 1. At baseline, carriers of damaging or unresolved *MC4R* mutations in group A were significantly younger (*p* = 0.019), had higher plasma TSH (*p* = 0.004) and lower plasma T4 (*p* = 0.023), compared to group B. The remaining variables showed no significant baseline differences. Additional adjustment for BMI did not change the results interpretation-wise for any of these comparisons (Table 1). Furthermore, exclusion of benign *MC4R* carriers from group B did not change these results (data not shown).

Applying correction for multiple testing, the difference in TSH levels stays borderline significant (*p* = 0.07), while baseline differences of age and plasma T4 are further away from global significance. Hence observed indications call for, and require, further replication.

### Effects of damaging or unresolved *MC4R* mutations following a structured lifestyle intervention

The number of patients diminished during the intervention, as some completed the intervention (achieved a BMI below the ~75th percentile for age and sex), moved away, requested to stop, or neglected appointments. In total, for 24 children from group A (24/30 = 80.0%) and 982 from group B (982/1179 = 83.3%) we were able to link eligible basic follow-up data and they were thus available for analyses of treatment effects on BMI SDS. Of note is that these numbers further decreased when analyzing blood pressure, blood and DXA variables (Table 2). The mean treatment duration was 1.1 years (range 0.5–3.7) for group A and 1.0 years (range 0.5–4.0) for group B.

When comparing phenotypic changes after lifestyle intervention between groups A and B, we found a significant difference in BMI SDS: group B decreased their BMI SDS, while group A did not (*p* = 0.005). Further, there was a significant difference in treatment response for HDL cholesterol, as group B did not change their HDL cholesterol, while group A decreased HDL cholesterol (*p* = 0.017). For the remaining traits, no significant differences in intervention response were seen between groups A and B. The same significant differences were observed after exclusion of benign *MC4R* carriers from group B (data not shown). Additional adjustment for BMI change did not substantially alter the results of the intervention responses (Table 2).

Applying correction for multiple testing, the difference in phenotypic changes of BMI SDS stays borderline significant (*p* = 0.08), while the difference in treatment response for HDL cholesterol was clearly outside global significance (*p* = 0.29). Hence, as above, observed indications all deserve further replication.

## Discussion

In our cohort of children and adolescents with overweight or obesity, we identified 30 carriers of damaging or unresolved *MC4R* mutations (2.5%). Our finding is similar to the previously reported prevalence of 2.5% in a sample of Danish men with juvenile-onset obesity [35], and is in accordance with other studies in samples of European individuals with obesity reporting prevalences between 0.2 and 6.3% [8, 11, 13, 15, 17, 18, 36, 44]. In comparison, the prevalence of pathogenic *MC4R* mutations has been estimated to be 0.15% in the general German population [36], and a similar prevalence would be expected in the Danish population. The specific mutation Y35X was identified in 12 children, indicating a relatively high prevalence in our cohort (1.0%). An increased prevalence of this mutation has previously also been described in Danish men with juvenile-onset obesity [35], in the German population [28], in a Dutch cohort of patients with obesity [45] and in ethnic Norwegian adults [46]. The GnomAD allele frequency of this mutation is 0.0001549% in European (non-Finnish) and has not been seen in other ethnicities (except one African). Our observation complements the previous suggestions that the Y35X mutation is a European founder mutation [35, 45, 46]. Although none of the included participants were born in known consanguineous families and one sibling from each identified sibling pair was excluded from the study, we cannot fully exclude the possibility of a family relationship between carriers of this haplotype.

Our classification of *MC4R* variants were based on the combined findings from several previous in vitro functional studies, and we have sought to evaluate *MC4R* variant functionality in terms of both cell-surface expression, ligand binding and signaling. Assessment of the receptor activation of MC4R has previously been focused on cyclic adenosine monophosphate (cAMP) signaling. More recently, MC4R has shown to activate also extracellular signal-regulated kinase (ERK) 1/2 in the mitogen-activated protein kinase pathway (reviewed in ref. [47]). As MC4R-induced activation of ERK1/2 is involved in mediation of food intake inhibition [48], disrupted ERK1/2 signaling may contribute to the obese phenotype observed in carriers of *MC4R* mutations. Our classification of the variants identified in the present study has therefore been based on functional studies examining both cAMP and ERK1/2 signaling, if available in the literature. The conservative means of classification of damaging *MC4R* variants in our study should ensure that the effects of true damaging mutations were not diluted. Nevertheless, we performed statistical analyses including children with damaging *MC4R* mutations and unresolved *MC4R* mutations to exclude the possibility that we may have excluded some damaging mutations from our analyses.

*MC4R* mutations have been associated with increased BMI, increased lean mass, increased linear growth, hyperphagia, and hyperinsulinemia; a condition collectively classified as MC4R deficiency or termed the MC4R syndrome [8, 44]. In the present study, we did not observe a baseline difference between carriers and noncarriers for any of these traits, which is in line with studies using similar sampling designs [11, 14, 16, 17]. Specifically, we did not observe a baseline difference for BMI and BFMI, an important result suggesting that among children with obesity, those with and without *MC4R*-induced obesity cannot be easily distinguished in the general population—a discovery that has been made by several other teams previously [4, 7, 49]. In contrast, among adults with obesity, carriers of *MC4R* mutations are heavier than their noncarrier counterparts [4, 7], indicating that *MC4R*-induced obesity leads to a more severe phenotype in adulthood. Lower SBP and/or DBP have been reported for individuals with loss-of-function *MC4R* mutations compared with noncarriers [10]. We did not find that MC4R mutation carriers had lower baseline SBP and/or DBP, in agreement with other studies [14]. Unlike previous studies [12, 16, 17], we found a difference in the baseline thyroid function status with a higher plasma concentration of TSH and a lower plasma concentration of T4 in *MC4R* carriers compared to noncarriers. Animal studies have shown alpha-melanocyte-stimulating hormone to have a direct effect on thyrotropin-releasing hormone-synthesizing neurons resulting in increased circulating concentrations of TSH, and as alpha-melanocyte-stimulating hormone is upregulated with decreasing MC4R activity [50, 51], this may explain our findings.

Summarized, the mutation carriers in our study did not display significant alterations in traits previously associated with MC4R deficiency [8, 44]. This discrepancy may be explained by our study sample only including heterozygous carriers. In the study by Farooqi et al. [8], 6 of the 29 carriers of *MC4R* mutations were homozygous, thus displaying a more severe phenotype. In addition, they did not include pubertal development stage in their analyses, and since puberty is known to influence BMI, height, circulating concentrations of insulin, and body fat distribution, our adjustments for pubertal development stage may partly explain why we did not identify the physiological profile previously associated with MC4R deficiency. Other studies [11, 14, 16, 17] have also failed to demonstrate the characteristics of MC4R deficiency, originally described by Farooqi et al. [8, 44].

After a minimum of 6 months of weight loss intervention, noncarriers had on average clearly decreased their BMI SDS, whereas carriers of *MC4R* mutations failed to do so; a change in intervention response that differed significantly between the two groups. This is in contrast to previous intervention studies finding carriers to lose weight to the same degree as noncarriers. In a cohort of Czech children and adolescents with obesity, carriers of *MC4R* mutations (*n* = 5) and noncarriers (*n* = 96) had a similar weight reduction following a 3 or 6 weeks weight reduction program [17]. A 1-year obesity intervention program in German children carrying *MC4R* mutations (*n* = 9) and noncarriers (*n* = 46) also showed similar weight reductions in carriers and noncarriers following intervention [16]. In both studies, no significant differences in other-examined traits were identified [16, 17]. Finally, a Spanish study described that among children with obesity, carriers of *MC4R* mutations (n = 8) achieved similar or greater reduction in BMI-SDS loss compared to noncarriers (*n* = 103) after a short 8-week lifestyle intervention [18]. However, only some of the carriers maintained the reduction in BMI SDS after 1 year of intervention [18].

Apart from a difference in BMI SDS, no significant other differences were seen in our study except for changes in HDL cholesterol, which was reduced in carriers compared to noncarriers. The apparent poorer response to a treatment program, which has proven effective in treating children with obesity in general [19, 27], may indicate that children carrying damaging *MC4R* mutations need a more explorative and likely more intensive program when aiming to reduce BMI SDS. In the Czech study [17], children were treated more intensively on an in-patient basis for the duration of the study period with daily physical activities and energy-restricted diets. In the German study [16], children were recruited after strict eligibility criteria where they were to complete an 8-week motivation phase prior to inclusion (47% dropout). They were then enrolled for a 3-month intensive phase with weekly consultations, followed by 9 months of monthly care. In comparison, our program consists of consultations every 1–2 months [19], and is thus of low intensity compared with the above-mentioned studies [16, 17]. Interestingly, voluntary exercise has been shown to attenuate the phenotypic characteristics of MC4R deficiency in mice [52, 53], further indicating a potential influence of physical activity on treatment outcome.

In a clinical trial enrolling the parents of the children with damaging *MC4R* mutations included in the present study, it was found that treatment with Glucagon-like peptide-1 receptor agonist liraglutide for 16 weeks induced an equal, clinically significant weight loss of 6% compared to matched controls [54]. These results indicate that the appetite-reducing effect of liraglutide is preserved in *MC4R* causal obesity and that liraglutide acts independently of the MC4R pathway.

In summary, results from various weight loss intervention programs indicate a need for personalized intervention based on *MC4R* genotype. Thus, it should be considered to screen children with obesity for *MC4R* mutations prior to treatment initiation to determine whether they may benefit from the standard program or if a more explorative program should be offered.

A strength of the present study was that carriers were identified from a relatively large group of children and adolescents with obesity. The children followed the treatment program without either patient or health care professionals having knowledge of their *MC4R* status, minimizing a potential bias in treatment response. Another strength was the thorough assessment of the functionality of each *MC4R* variant and pediatrician-assessed pubertal stage.

Our study has some limitations: primarily, the study is not a clinically controlled and randomized study; rather it is an intention-to-treat program, which has previously proven effective in weight reduction [19, 27]. In addition, patients were only genetically screened for *MC4R* mutations, and some participants may thus have other forms of monogenic obesity. We selected potential mutations using the ExAC database, which in the meantime has been replaced by the larger gnomAD database (https://gnomad.broadinstitute.org). Furthermore, as this is a clinical study in a treatment setting, the cohort number was derived from and limited by an inclusion period. Although analyses on the intervention response were performed on all available measures for all individuals meeting the study-specific inclusion criteria, we were not able to include data on all carriers. This was primarily due to diminishing sample numbers during the intervention and to our criterion of a maximum of 60 days between anthropometric measurements and blood samples or DXA scan. Finally, the measurements of FFMI, BFMI, and BMD were not expressed as SD scores and further, they were performed on one type of DXA scanner in the beginning of the treatment period and on another for the remainder of treatment time. The scanners were from the same vendor, and were calibrated daily, but even though we adjusted our analysis concerning DXA measures for type of scanner, our consistency of estimates of body composition values and changes might have been slightly affected.

We conclude that in a sample of Danish children and adolescents with obesity, we found a prevalence of damaging or unresolved *MC4R* mutations of 2.5% and showed that at baseline carriers of *MC4R* mutations had higher fasting plasma concentrations of TSH, and lower fasting plasma concentrations of T4 than noncarriers. When following a validated childhood obesity treatment program for a mean duration of 1 year, carriers of damaging *MC4R* mutations exhibited no reduction in their BMI SDS—in contrast to noncarriers. Furthermore, HDL cholesterol was reduced in carriers, a response significantly different from that of noncarriers. Our findings indicate a need for individualized and explorative interventions based on *MC4R* genotype.

## Data Availability

Relevant data for the present study are within the article. If you wish to see additional data, the authors confirm that, for approved reasons, some access restrictions apply to the data underlying the findings. Data are available from the Novo Nordisk Foundation Center for Basic Metabolic Research, whose authors may be contacted at torben.hansen@sund.ku.dk.
